# Predicting long-term disease control in transplant-ineligible patients with multiple myeloma: impact of an MGUS-like signature

**DOI:** 10.1038/s41408-019-0176-x

**Published:** 2019-03-18

**Authors:** Paula Rodríguez-Otero, María Victoria Mateos, Joaquín Martínez-López, Miguel-Teodoro Hernández, Enrique M. Ocio, Laura Rosiñol, Rafael Martínez, Ana-Isabel Teruel, Norma C. Gutiérrez, Joan Bargay, Enrique Bengoechea, Yolanda González, Jaime Pérez de Oteyza, Mercedes Gironella, Jorge M. Nuñez-Córdoba, Cristina Encinas, Jesús Martín, Carmen Cabrera, Luis Palomera, Felipe de Arriba, María Teresa Cedena, Noemí Puig, Albert Oriol, Bruno Paiva, Joan Bladé, Juan José Lahuerta, Jesús F. San Miguel

**Affiliations:** 1 Clínica Universidad de Navarra, CIMA, IDISNA, CIBERONC, Pamplona, Spain; 2 Complejo Asistencial Universitario de Salamanca, Instituto de Investigación Biomédica de Salamanca, Salamanca, Spain; 3 Hospital Universitario 12 de Octubre, Instituto de Investigación 12 de Octubre, CIBERONC, Madrid, Spain; 4 Hospital Universitario de Canarias, Santa Cruz de Tenerife, Spain; 5 Hospital Clinic I Provincial, Institud’Investigacions Biomèdiques August Pi i Sunyer (IDIBAPS), Barcelona, Spain; 6 Hospital Clínico San Carlos, Madrid, Spain; 7 Hospital Clínico de Valencia, Valencia, Spain; 8 Hospital Son Llatzer, Palma de Mallorca, Spain; 9 Hospital de Donostia, San Sebastian, Spain; 10 Institut d’Oncologia Dr. Josep Trueta, Girona, Spain; 11 Hospital de Madrid Sanchinarro, Universidad CEU San Pablo, Madrid, Spain; 12 Hospital Vall d’Hebron, Barcelona, Spain; 13 Research Support Service, Central Clinical Trials Unit, University Clinic of Navarra, Pamplona, Spain; 14 Hospital General Universitario Gregorio Marañón, Instituto de Investigación Sanitaria Gregorio Marañón (IiSGM), Madrid, Spain; 15 Hospital General Virgen del Rocío, Sevilla, Spain; 16 Hospital San Pedro de Alcántara, Cáceres, Spain; 17 Hospital Clínico Universitario Lozano Blesa, Zaragoza, Spain; 18 Servicio de Hematología y Oncología Médica, Hospital Universitario Morales Meseguer, IMIB-Arrixaca, Universidad de Murcia, Murcia, Spain; 19 Insitut Català d’Oncologia, Institut Josep Carreras, Hospital German Trias i Pujol, Badalona, Barcelona, Spain

## Abstract

Disease control at 5 years would be a desirable endpoint for elderly multiple myeloma (MM) patients, but biomarkers predicting this are not defined. Therefore, to gain further insights in this endpoint, a population of 498 newly diagnosed transplant-ineligible patients enrolled in two Spanish trials (GEM2005MAS65 and GEM2010MAS65), has been analyzed. Among the 435 patients included in this post-hoc study, 18.6% remained alive and progression free after 5 years of treatment initiation. In these patients, overall survival (OS) rate at 10 years was 60.8% as compared with 11.8% for those progressing within the first 5 years. Hemoglobin (Hb) ≥ 12 g/dl (OR 2.74, *p* = 0.001) and MGUS-like profile (OR 4.18, *p* = 0.005) were the two baseline variables associated with long-term disease-free survival. Upon including depth of response (and MRD), Hb ≥ 12 g/dl (OR 2.27) and MGUS-like signature (OR 7.48) retained their predictive value along with MRD negativity (OR 5.18). This study shows that despite the use of novel agents, the probability of disease control at 5 years is still restricted to a small fraction (18.6%) of elderly MM patients. Since this endpoint is associated with higher rates of OS, this study provides important information about diagnostic and post-treatment biomarkers helpful in predicting the likelihood of disease control at 5 years.

## Introduction

Treatment of multiple myeloma (MM) has significantly improved in the last decade but the outcome remain significantly poorer in elderly patients^1^. Thus, while approximately 34–60% of transplant-eligible cases remain progression free at 5 years^2–4^ and this has resulted in unprecedent rates (10–40%) of 10-year overall survival (OS)^2–4^; the percentage of elderly patients reaching disease control at 5 years and the characteristics of such patients is less well established. Moreover, in the non-transplant-eligible population, definition of long-term disease control could be different, since patients that are free from relapse beyond 5 years, could eventually die for reasons other than progressive disease. Thus, achieving disease control beyond 5 years would be a most relevant endpoint, since elderly patients usually have fewer options for subsequent lines of therapy, and a prolonged first response will translate not only into a longer survival but also in improved quality of life.

Several studies have searched for relevant prognostic factors associated with prolonged survival in elderly MM. Thus, in addition to a fit status, according to geriatric assessment^5^, and absence of high-risk cytogenetic abnormalities (HR CAs)^6^, both the International Staging System (ISS) and the Revised-ISS (R-ISS)^7,8^ clearly identified that ISS 1 and R-ISS 1 patients enjoy a significantly longer progression-free survival (PFS) and OS. Additional conventional factors such as normal hemoglobin (Hb), creatinine, and beta-2-microglobulin (B2M) levels, age below 80 years and normal lactate dehydrogenase (LDH) levels, as well as, low percentage of S-phase bone marrow (BM) plasma cells (PCs), have been identified as predictors for longer PFS and OS^9–11^. Nevertheless, none of these studies have specifically focused on the identification of the characteristics of the patients' cohort that achieve a long-term disease control (PFS > 5 years).

Depth of response is also associated with prolonged survival in elderly patients. Thus, complete response (CR) defined by conventional criteria translates into longer PFS and OS^12–14^. More recently, it has been shown that the achievement of sustained minimal residual disease negativity clearly predict for a longer PFS and OS in non-transplant candidate patients^15^. However, there is a small subset of MM patients that despite persistence of the disease enjoy long-term disease control, underlying the need of new tools to identify these long-term survivors.

Interestingly, it has been reported that patients with a previous diagnosis of monoclonal gammopathy of unknown significance (MGUS) or smoldering MM (SMM) have a more prolonged survival^16^. However, although it is well known that all MM evolve from a prior MGUS, in most patients presenting with active disease no information on a previous MGUS-status is available. Noteworthy, both the Arkansas^17^ and the Spanish groups^18^ have reported, by using gene expression profiling (GEP) and multiparameter flow cytometry (MFC) techniques, respectively, that an MGUS signature, associated with low-risk features and superior survival, can be identified in a fraction of patients with active MM.

Here, we evaluated a large series of 498 transplant-ineligible MM patients enrolled in two consecutive Spanish clinical trials, treated with first-generation novel agent-based combinations and with mature follow-up. Our aims were to (i) determine the percentage of elderly patients with disease control at 5 years after treatment initiation, (ii) analyze whether this translates into a substantial benefit in OS, and (iii) define an optimal set of biomarkers to estimate the odds of achieving disease control at 5 years.

## Patients and methods

### Study design

Four hundred ninety-eight newly diagnosed elderly and symptomatic MM patients (aged 65 years or older) were included in two prospective Spanish trials: GEM2005MAS65 and GEM2010MAS65^19–22^. Patients with a follow-up shorter than 5 years (*n* = 22) or who died without progression (*n* = 41) were censored from the analysis and, accordingly, 435 patients were evaluated in this post-hoc study. All patients were diagnosed according to the IMWG criteria^23^. Data cut-off was 20 February 2018. All patients provided written informed consent before screening. Data were monitored by an external contract research organization and centrally re-assessed. Median follow-up was 92.6 months (range: 0.3–132.9 months) and 61.7 (range: 1.6–81.1 months) for the GEM2005MAS65 and the GEM2010MAS65 studies, respectively. The endpoint of this study was defined as time to progression at 5 years or longer since diagnosis, in patients with a follow-up of at least 5 years.

The GEM05MAS65 trial included 259 patients randomized to receive upfront induction treatment with either bortezomib, melphalan, and prednisone (VMP), or bortezomib, thalidomide, and prednisone (VTP)^16^. VMP induction therapy consisted of six cycles: one cycle of intravenous bortezomib given twice per week for 6 weeks (1.3 mg/m^2^ on days 1, 4, 8, 11, 22, 25, 29, and 32), plus oral melphalan 9 mg/m^2^ and prednisone 60 mg/m^2^ on days 1–4, followed by five cycles of bortezomib once per week for 5 weeks (1.3 mg/m^2^ on days 1, 8, 15, and 22) plus the same doses of melphalan and prednisone. VTP induction therapy consisted of the same schedule of bortezomib and prednisone plus oral, continuous, thalidomide at a dose of 100 mg per day instead of melphalan. Patients from each arm completing the six induction cycles were then randomly assigned to maintenance therapy with either bortezomib and thalidomide (VT) or bortezomib and prednisone (VP). Maintenance consisted of one conventional cycle of bortezomib (1.3 mg/m^2^ on days 1, 4, 8, and 11) every 3 months, plus either oral prednisone (50 mg every other day) or oral thalidomide (50 mg per day), for up to 3 years.

In the GEM010MAS65, 239 patients were randomly assigned 1:1 to receive both VMP and lenalidomide in combination with low-dose dexamethasone (Rd) in a sequential or alternating manner, for 18 cycles^17^. VMP therapy comprised nine cycles: one 6-week cycle of IV bortezomib using a conventional twice-weekly schedule (1.3 mg/m^2^ on days 1, 4, 8, 11, 22, 25, 29, and 32), plus oral melphalan (9 mg/m^2^) and oral prednisone (60 mg/m^2^) on days 1–4, followed by eight 4-week cycles of once-weekly IV bortezomib (1.3 mg/m^2^ on days 1, 8, 15, and 22) plus the same doses of melphalan and prednisone. Rd treatment consisted of nine cycles of oral lenalidomide 25 mg daily on days 1–21 of each 28-day cycle plus oral dexamethasone 40 mg weekly, on days 1, 8, 15, and 22.

The following characteristics, documented at diagnosis, were analyzed: age, gender, Hb, serum creatinine levels, B2M, albumin, PC infiltration, LDH, type of monoclonal component, presence of urine light chains, extramedullary disease, clinical staging according to the ISS and R-ISS, plus chromosomal abnormalities defining high-risk patients: *t*(4;14) and/or *t*(14;16) and/or del(17p).

In addition, an immunophenotypic algorithm to determine if patients had MGUS-like vs MM-like profiles was used by comparing the relative frequency of BM PCs plus the percentage of clonal and normal PCs within the whole BM PC compartment for each patient, plotted against a database containing information on the same three parameters from a total of 1774 patients, including 497 MGUS and 1277 newly diagnosed MM enrolled in the GEM2000 (*N* = 486) plus GEM2005MENOS65 (*N* = 330) protocols for transplant-eligible cases, and the GEM2005MENOS65 (*N* = 239) plus GEM2010MAS65 (*N* = 222) protocols for transplant-ineligible patients. Based on those three parameters and the automatic population separator (APS) representation of the Infinicyt software (Cytognos, Salamanca, Spain), two reference groups corresponding to MGUS and MM emerged; each patient falling into the MGUS reference being classified as MGUS-like (Supplementary Figure 1)^18^.

Fluorescence in situ hybridization (FISH) on CD138-purified PCs and immunophenotyping were performed centrally at diagnosis following standard procedures^6^. Responses were evaluated according to the European Group for Blood and Marrow Transplantation^24^ and IMWG^25^ criteria.

### Statistical analysis

The endpoint of this study was defined as time to progression at 5 years or longer since diagnosis, in patients with a follow-up of at least 5 years.

The baseline characteristics of the patients are presented as mean and standard deviation (SD) for continuous variables and as proportions for categorical variables. Univariate analysis was performed using the chi-squared or Fisher’s exact test, as appropriate, for independent variables. Multivariate analysis was performed using a forward-stepwise model. Variable selection to be tested in the multivariate analysis was carried out using an automated selection method (forward-stepwise). Two significance levels were specified in the forward-stepwise procedure: 10% for predictor addition to the model, and 20% for predictor removal from the model. A first model including all the baseline variables was performed and a second model including depth of response based on conventional and minimal residual disease evaluation was conducted using the same criteria. In both models, the R-ISS was included instead of ISS, LDH, and HR CAs in order to avoid redundancy. A landmark analysis at 6 and 12 months has been performed including all patients. Crude survival analysis was assessed using log-rank test. Kaplan–Meier survival analysis was carried out for each group category. All statistical analyses were carried out using Stata 12.1.

## Results

The median PFS for the whole population included in the GEM05MAS65 and GEM2010MAS65 (*n* = 498) was 31.4 month (95% confidence inerval (CI), 28.28–34.36), being 28.3 months (95% CI, 24.8–32.9) and 34.3 months (95% CI, 29.6–39.1) in each trial, respectively. The median OS was 57.3 months (95% CI, 49.1–65.4), being 51.6 months (95% CI, 42.9–61.1) and 68.2 months (95% CI, 49.1–NR) for patients included in the GEM05MAS65 and GEM2010MAS65 trials, respectively.

**Fig. 1:**
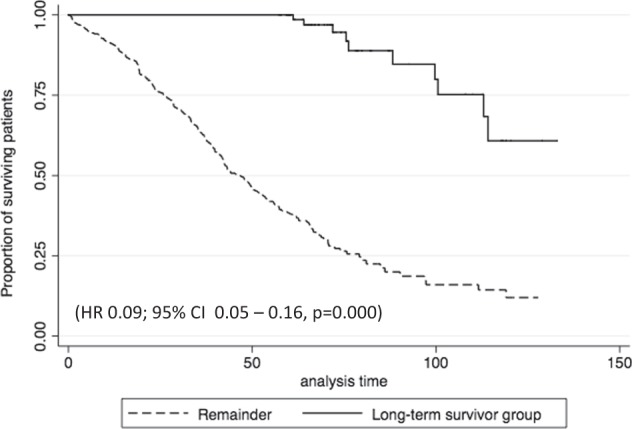
**Kaplan–Meier curves of overall survival in long-term survivor group as compared with the remainder**

Four hundred thirty-five newly diagnosed transplant-ineligible MM patients were analyzed in this post-hoc study. Eighty-one out of 435 patients (18.6%) were alive and without disease progression during the first 5 years of initiating treatment and this group of patients has been identified as the long-term disease-free survivor group. The remaining 354 patients have progressed in the first 5 years after the diagnosis. The proportion of patients alive at 10 years in the long-term disease-free survivor group was 60.8% (95% CI, 35.4%–78.8%) as compared with 11.8% (95% CI, 5.9%–19.8%) (*p* < 0.001), respectively (Fig. 1).

Results of the univariate analyses are summarized in Table 1. Baseline variables significantly associated with long-term PFS were, ISS 1 (35% vs 22.3%; *p* = 0.036), R-ISS 1 (30% *vs* 16.6%, *p* = 0.002), Hb ≥ 12 g/dl (34.6% vs 17.8%, *p* = 0.001), normal LDH (97.5% vs 87.6%, p = 0.009), and absence of HR CAs (91% vs 80.9%, *p* = 0.04). Moreover, patients in the long-term disease-free survivor group presented more frequently an MGUS-like profile (15.6 % vs 3.7%, *p* < 0.001). In addition, quality of response measure by both the proportion of complete remissions (CRs) (58% vs 33.3%, *p* = 0.001), and MRD-negative cases (41.9% vs 9.9%, *p* < 0.001) were also higher in the long-term PFS group.

**Table 1:** Distribution of the main characteristic between the long-term survival group and the remainder population (univariate analysis)

Variable	Long-term survival, *n* (%)	Remainder, *n* (%)	*p*-Value
Age	*n* = 81	*n* = 354	*p* = n.s.
<75 years	56 (69.1)	224 (63.2)	
Gender	*n* = 81	*n* = 354	*p* = n.s.
Male	46 (56.8)	178 (50.3)	
Protocol	*n* = 81	*n* = 354	*p* = n.s.
GEM05MAS65	44 (54.3)	206 (58.2)	
GEM2010MAS65	37 (45.7)	148 (41.8)	
Durie stage	*n* = 81	*n* = 352	*p* = n.s.
I	12 (14.8)	29 (8.2)	
II	42 (51.8)	168 (47.8)	
III	27 (33.3)	155 (44)	
ISS	*n* = 80	*n* = 350	*p* = 0.036
ISS 1	28 (35)	78 (22.3)	
ISS 2	33 (41.2)	151 (43.1)	
ISS 3	19 (23.8)	121 (34.6)	
R-ISS	*n* = 80	*n* = 350	*p* = 0.002
R-ISS 1	24 (30)	58 (16.6)	
R-ISS 2	55 (68.8)	256 (73.1)	
R-ISS 3	1 (1.2)	36 (10.3)	
Hemoglobin (g/dl)	*n* = 81	*n* = 354	*p* = 0.001
Hb ≥ 12 g/dl	28 (34.6)	63 (17.8)	
Creatinine (mg/dl)	*n* = 81	*n* = 354	*p* = n.s.
Creat < 1.1	54 (66.7)	242 (68.4)	
LDH	*n* = 81	*n* = 348	*p* = 0.009
Normal	79 (97.5)	305 (87.6)	
Cytogenetics	*n* = 75	*n* = 335	*p* = 0.04
Standard risk	68 (91)	271 (80.9)	
Response	*n* = 81	*n* = 327	*p* = 0.001
CR (sCR + CR IF-)	47 (58)	109 (33.3)	
VGPR	27 (33.3)	161 (49.2)	
PR	4 (4.9)	45 (13.8)	
SD	3 (3.7)	11 (3.4)	
PD	0 (0)	1 (0.3)	
BM signature	*n* = 77	*N* = 323	*p* < 0.001
MGUS-like profile	12 (15.6)	12 (3.7)	
MM-like profile	68 (84.4)	311 (96.3)	
MRD	*n* = 81	*n* = 354	*p* < 0.001
Negative	35 (41.9)	35 (9.9)	

*n.s.* not statistically significant, *MRD* minimal residual disease, *CR* complete response, *sCR* stringent complete response, *CR IF-* complete response with negative immunofixation, *VGPR* very good partial response, *PR* partial response, *SD* stable disease, *PD* progressive disease, *Hb* hemoglobin, *Creat* creatinine, *ISS* International Staging System, *R-ISS* revised International Staging System, *LDH* lactate deshydrogenase

The multivariate logistic regression analysis showed that the probability of long-term disease control was associated with two independent baseline factors (Table 2a): the presence of an Hb ≥ 12 g/dl (OR 2.74; 95% CI, 1.53–4.89, *p* = 0.001), and the presence of an MGUS-like profile in the BM PCs (OR 4.18; 95% CI, 1.5–11.3, *p* = 0.005). When incorporating depth of response and patients’ MRD status into the model, three variables were found to be independently associated with the probability of 5-year PFS (Table 2b): the presence of an MGUS-like profile (OR 7.48; 95% CI, 2.46–22.74, *p* < 0.001); the MRD negativity (OR 5.18; 95% CI, 2.77–9.69, *p* < 0.001), and the Hb ≥ 12 g/dl (OR 2.27; 95% CI, 1.22–4.22, *p* = 0.009). Moreover, after performing a landmark analysis at 6 and 12 months including all patients (*n* = 498), the three variables significantly associated with long-term survival remain the same (Hb > 12, MGUS-like profile and MRD negativity).

**Table 2a:** Multivariate analysis including baseline variables

	Odds ratio	95% CI	*p*-Value
MGUS-like profile	4.18	1.55–11.26	0.005
Hb ≥ 12 g/dl	2.74	1.53–4.89	0.001

*MGUS* monoclonal gammopathy of unknown significance, *Hb* hemoglobin, *95% CI* 95% confidence interval

**Table 2b:** Multivariate analysis including depth of response measured by complete response and MRD negativity rates

	Odds ratio	95% CI	*p-*Value
MGUS-like profile	7.48	2.46–22.74	0.000
Hb ≥ 12 g/dl	2.27	1.22–4.22	0.009
MRD negativity	5.18	2.77–9.69	0.000

*MGUS* monoclonal gammopathy of unknown significance, *Hb* hemoglobin, *MRD negativity* minimal residual disease negativity detected by flow cytometry

Focusing on the 24 patients with an MGUS-like signature, 50% of these patients displayed a long-term disease-free survival, as compared with 17.3% of the remaining MM patients. The proportion of patients alive at 10 years with an MGUS-like profile was 40.5% (95% CI, 9.4–70.7) as compared with 20.3% (95% CI, 12.7–29.2) in the MM-like population (*p* = 0.07). Similarly, for PFS, the proportion of patients alive and without disease progression at 10 years in the MGUS-like group was 40.2% (95% CI, 20.5–59.1) as compared with 5.7% (95% CI, 2.8–10.1) among the MM-like patients (*p* = 0.001). Disease response has been evaluated in 21 out of 24 patients with MGUS-like profile and 90% of the patients achieved a favorable response (10 CR and 9 VGPR). No differences in outcome were observed between VGPR and CR cases (*p*-value for OS 0.78) among the MGUS-like profile patients. Interestingly, as suggested by the multivariate analysis, the impact of the MGUS-like signature on long-term survival is independent on the MRD status, since only 16.7% of these patients achieved MRD negativity, as compared with 46.15% patients in the long-term survival group with a MM-like profile (*p* = 0.057).

## Discussion

In the transplant setting, it has been clearly shown that improving the median PFS beyond 5 years resulted in median OS of 8–12 years^2–4^. However, it is not so well established if disease control at 5 years in elderly MM has a similar impact in OS. Indeed, in this setting it may be even more relevant, since many elderly patients will only receive 2–3 lines of therapy, and accordingly, a prolonged initial response could translate both in a prolonged survival, similar to that of elderly healthy individuals, and an improved quality of life. The definition of long-term disease control used in the present study (PFS beyond 5 years) is quite challenging since it is equivalent to the median OS reported in the VISTA^26^ and FIRST trials^27^ based on VMP (OS: 56 months) or continuous Lenalidomide-dexamethasone (OS: 59 months) treatments, respectively. Upon considering VRD as a new standard of care, the median PFS reported for patients above the age of 65 (35 months) is clearly inferior to the 5 years here proposed^28^. Taken together, it should be assumed that despite substantial improvement in the treatment of MM, the proportion of elderly patients achieving long-term disease control (i.e.: > 5 years PFS) is limited and factors identifying this population upfront are lacking.

In our series, only 18.6% out of 435 elderly patients entered in this category, and 61% of them achieved an OS of more than 10 years, accordingly they will reach a life expectancy similar to the normal population. As expected, this patients' cohort is characterized by the presence of low-risk clinical features (R-ISS 1, normal Hb, and LDH). The ISS, particularly the revised version (R-ISS), that include the cytogenetic information, is a powerful tool in discriminating risk subgroups, but their predictive value for 5-year disease control is not established. By contrast, the emergence of normal Hb levels as a strong predictor of long-term disease control was surprising and could reflect a lower BM infiltration and potentially a better tolerance to treatment. But the most relevant biomarker for predicting long-term disease control upfront was the presence of an MGUS-like signature defined by automated MFC and characterized by a low number of bone BM PCs together with a relative preservation in the percentage of normal vs clonal PCs within the whole BM PC compartment. The favorable outcome of patients with an MGUS-like profile was first shown by the Arkansas group using a gene expression signature based on 52 genes^17^. From 214 patients with MM, 27% were found to have an MGUS-like signature, and it was associated with low-risk clinical and molecular features and superior survival. Moreover, the MGUS-like signature was also seen in PCs from 15 of 20 patients surviving >10 years after auto-transplantation. By using MFC, Paiva et al.^18^ had shown that 59 out of 689 transplant candidate MM patients (8%) displayed an MGUS-like profile. Despite achieving similar CR rates after high-dose therapy vs other MM patients, MGUS-like cases had very long time to progression and OS (~60% at 10 years; *p* < 0.001). Although, MGUS-like signature does not necessarily drive a long time to progression, our data indicate that this biomarker can contribute to identify a subset of symptomatic MM patients with an excellent outcome, independently of the depth of response. Overall, these data could also imply that treating patients before massive disease dissemination and niche occupancy in BM confers favorable prognosis.

Although the depth of response is not predictable at the time of diagnosis, it was shown to be a significant predictive marker of long-term disease control when measured with conventional methods (CR) or, particularly, by MFC (MRD negativity). Noteworthy, our study highlights that the prognostic impact of a MGUS-like signature, is even more relevant than to achieve MRD negativity for predicting long-term disease control (HR of 7.09 and 4.87, respectively). Although MRD is becoming a most valuable treatment endpoint and a surrogate marker for survival, the fact that MGUS-like and MRD are independent confirms that for some patients the achievement of an MRD-negative status is not imperative for attaining long-term disease control.

In summary, we identified that the combination of three biomarkers (normal Hb, MGUS-like signature, and MRD negativity) can help to define elderly MM patients achieving long-term disease control. Although these findings will be validated in our next GEM2017FIT trial for elderly newly diagnosed MM patients, our results show that the presence of a MGUS-like signature in the BM at diagnoses is the most powerful predictor for long-term disease-free survival, becoming an important prognostic biomarker.

## Supplementary information

Supplementary Figure 1

Reproducability checklist
