# Supplementary material for: Predicting long-term disease control in transplant-ineligible patients with multiple myeloma: impact of an MGUS-like signature
Source: Blood Cancer J. 2019 Mar 18;9(4):36. doi: 10.1038/s41408-019-0176-x (PMC6423121; doi:10.1038/s41408-019-0176-x)
Supplement: Supplementary file 1 — Supplementary Figure 1 [file 41408_2019_176_MOESM1_ESM.pptx]

## Slide 1
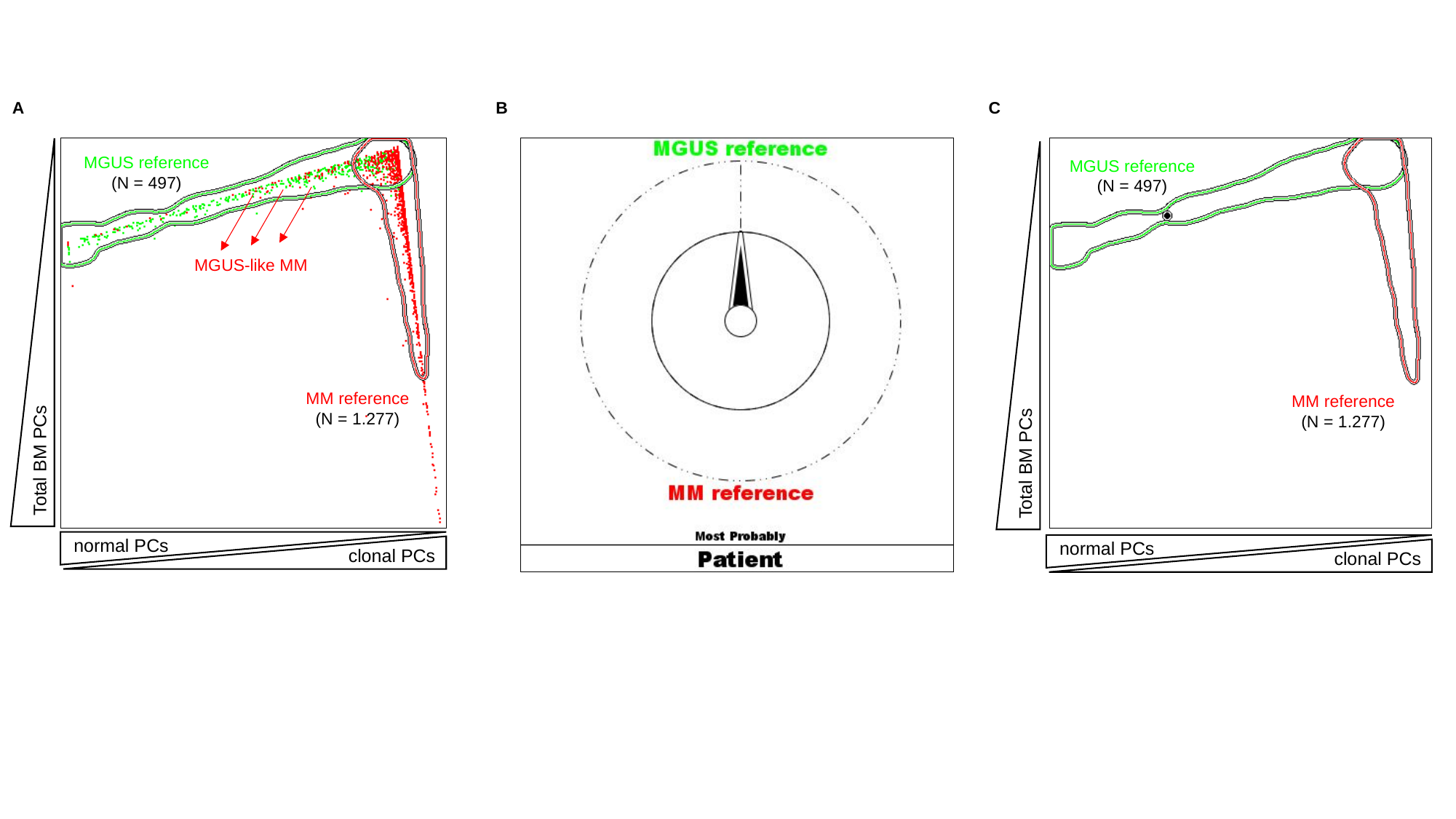

A
B
C
MGUS reference
(N = 497)
MGUS reference
(N = 497)
MGUS-like MM
MM reference
(N = 1.277)
MM reference
(N = 1.277)
Total BM PCs
Total BM PCs
normal PCs
normal PCs
clonal PCs
clonal PCs
